# Antioxidant activity, total polyphenol content and methylxantine ratio in four materials of *Theobroma cacao* L. from Tolima, Colombia

**DOI:** 10.1016/j.heliyon.2022.e09402

**Published:** 2022-05-10

**Authors:** Juan G. Borja Fajardo, Heidi B. Horta Tellez, Giann C. Peñaloza Atuesta, Angélica P. Sandoval Aldana, Jonh J. Mendez Arteaga

**Affiliations:** a Interdisciplinary Research Group on Tropical Fruit Cultivation, Faculty of Agronomic Engineering, University of Tolima, Cl. 42 #1b-1, Ibagué, Colombia; b Natural Products Research Group, Department of Chemistry, Faculty of Sciences, University of Tolima, Cl. 42 #1b-1, Ibagué, Colombia

**Keywords:** HPLC, Methylxanthines, Morphological, Theobromine, Polyphenols

## Abstract

The International Cocoa Organization recognized Colombian cocoa as "fine aroma," but in recent years, clone CCN 51 has grown in popularity, widely due to its high yield. The Tolima department is the fourth producer of cacao in Colombia, but there is a lack of knowledge of the chemical properties of regional cocoa genotypes. The aim of this study was to evaluate the morphological, antioxidant activity, total polyphenol content and the methylxanthines ratio of four regional genotypes (UTLP02, UTVE01, UTGC01 and UTLM02) of *Theobroma cacao* L. from Tolima, Colombia. The universal clone of CCN51 was used as control. The highest values for the qualitative descriptors were obtained by the variants UTVE01 and CCN51 with FRAP and TPC ranging from 44.51 ± 0.90 to 106.77 ± 5.21 mg GAE/g and 27.13 ± 0.14 to 52.12 ± 4.71 mmol TE/g respectively. The genotypes with the highest values for FRAP and TPC were UTGC01 and CCN51. According to the methylxanthine ratio, UTVE01 was classified as Criollo, while UTLM02, UTGC01 and UTLP02, CCN51 are Trinitario and Forastero, respectively. Although CCN51 is considered a remarkable material in terms of productivity, the genotypes evaluated present good yields and interesting values of TPC and antioxidant activity, making them promising trees in local breeding programs.

## 1 Introduction

Beans from the cocoa tree, *T. cacao* L*.,* are the raw material to produce chocolate and its derived products. It is an important crop for developing countries in Africa, Latin America and Asia (Samaniego et al*.,* 2020). Conventionally T. Cacao L. has been classified into three main groups, Forastero, Criollo and Trinitario, being the last a hybrid between the two first genetic groups (Scollo et al., 2020). Forastero is responsible for about 95% of the total worldwide production, due to its high productivity and resistance to diseases and pests (Jaramillo Flores, 2019). According to its quality, Criollo and Trinitario varieties generally produce “fine or flavour cocoa,” while Forastero clones usually produce bulk, basic or ordinary cocoa with some exceptions (Devy et al., 2019).

The main components of cocoa beans are fat, carbohydrates, proteins, vitamins, and minerals. Besides these metabolites, the cocoa is rich in polyphenols and methylxanthines (theobromine and caffeine), with varying concentrations depending on origin, genotype, soil factors, climatic conditions, and post-harvest management (Brunetto et al., 2007). The polyphenols in *T. cacao L*. can be classified in three major groups catechins or flavan-3-ols (37%), anthocyanins (4%), and proanthocyanidins (58%) (Laličić-Petronijević et al., 2016). The main flavan-3-ols is (-)-epicatechin with up to 35% of polyphenol content (Wollgast and Anklam, 2000), other phenolic compounds in smaller amounts are (-)-epigallocatechin, quercetin, chlorogenic acid, clovamide, naringenin, luteolin, biapigenin, ferulic and gallic acids, have also been found (Rodriguez-Carrasco et al., 2018). These metabolites have received special attention due to their role in prevention or management of diseases believed to be caused by oxidative stress such as several types of cancer, as well as cardiovascular and cerebrovascular diseases (Rimbach et al., 2009; Martín and Ramos, 2017).

Theobromine (3,7-dimethylxanthine) is the main alkaloid in cocoa (∼3.7% on a fat-free basis) followed by caffeine (1,3,7-trimethylxanthine) in smaller amounts (∼2%) and traces of theophylline (EFSA, 2008). The TB/CF ratio varies depending on the cocoa genotype, and therefore can be used to classify cocoa genotypes objectively (Davrieux et al., 2007). The limits of the TB/CF ratio for each type are from 3, 3 to 9, and more than 9 for Criollo, Trinirario and Forastero, respectively (Carrillo et al., 2014; Brunetto et al., 2007). Criollo beans contain 2/3 less polyphenols than Forastero). These compounds mainly act as stimulants of the central nervous system and, along with the polyphenols, are related with the bitterness and astringency taste found in chocolate (Afoakwa et al., 2008).

The geographic location of Colombia and the diversity of ecological conditions leads to a cocoa production with high variability in its chemical profile, affecting the sensory attributes of the final products (Carrillo et al., 2014). Colombia has made important advancements in the selection and development of new cocoa genotypes that are both high-yield and disease-resistant (Rodriguez-Medina et al., 2019). However, in certain regions, like Tolima, the morphological and chemical characterization of new potential genotypes of Criollo and Trinitario is incipient. Therefore, it is important to find native genotypes in the producer areas, so as to avoid their loss by the incorporation of new clones with higher yields and resistances but, with less aromatic and flavour profile. The aim of this study was to evaluate the morphological, antioxidant activity, total polyphenol content and methylxanthines ratio of four regional genotypes (UTLP02, UTVE01, UTGC01 and UTLM02) of *T. cacao* L. from Tolima, Colombia.

## 2 Materials and methods

### 2.1 Chemical and reagents

All solvents were of analytical or HPLC grade and supplied by Merck Chemicals (Darmstadt, Germany). Deionized water was obtained with a Wasserlab Autwomatic. Folin-Ciocalteu´s reagent was obtained from Panreac, Trolox (6-Hydroxy-2,5,7,8-tetramethylchroman-2-carboxylic Acid), TPTZ (2,3,5-Triphenyltetrazolium chloride) and the standard of theobromine-caffeine were obtained from Sigma Aldrich, St. Louis, MO, USA.

### 2.2 Sample collection and vegetal material selection

The assessment was carried out in different agro-ecological zones where cocoa is grown in the municipality of Chaparral (3°43′25″N, 5°29′05″O) and its surroundings. The farms and trees that were selected presented a continuous production process between January and December 2018. The four genotypes chosen UTLP02 (3°42′20.657″N, 75°35′46.363″O), UTVE01 (3°39′29,43″N, 75°36′19,40″O), UTGC01 (3°44′38.375″N, 75°32′23.575″O) and UTLM02 (3°47′16,25″N, 75°26′25,32″O) are found in different agroclimatic zones of the municipality between 770 to 1200 m above mean sea level with an average temperature 30 °C. 15 cocoa pods were collected of each genotype, using an average of 450 beans for the study. The CCN51 clone was collected in the same region and period.

### 2.3 Morphological characterization

The morphological evaluation for cocoa genotypes was performed based on quantitative descriptors to generate a profile for each material in triplicate, according to protocols published by Philips et al. (2012) (Table 1).

**Table 1:** Morphological descriptors evaluated for the cocoa genotypes.

Qualitative descriptors	Abbreviation
Weight of the fruit	WEI_FRU
Length of the fruit	LON_FRU
Diameter of the fruit	DIA_FRU
Weight of the seed	WEI_SEM
Number of seeds	NUM_SE
Seed index	SE_IND
Weight of the husk	WEI_HUS
Shell thickness	SHE_THI
Ratio length/diameter of the seed	L/D

### 2.4 Sample preparation

The beans were extracted according to Carrillo, Londoño & Gil (2013), with some modifications. The husk was removed manually and the beans were dried in a drying stove for 96 h at 40 °C, 0.1 g of crushed cocoa were defatted using n-hexane with vortex agitation for 10 min. The procedure was repeated three times. The degreased material was extracted with 1.5 ml of an aqueous solution of 2-propanol (60% v/v, pH 9.0) for 1 h in an ultrasonic bath (Elma, Germany, 37kHz). Afterward, the mixtures were decanted and filtered through a nylon membrane (0.45 μm, Whatman). Finally, the extracts were stored in ultra-freeze at -80 °C until analysis.

### 2.5 Total polyphenols content (TPC)

TPC was assessed by UV–vis spectrophotometry according to Singleton et al. (1999) with slight modifications. 0.25 mL of the extract, previously diluted, was mixed with 0.25 ml of the Folin-Ciocalteu reagent and 2.0 mL of distilled water. After 3 min at room temperature (25 °C) 0. 25 of sodium carbonate 20% w/v (Na_2_CO_3_) solution was added and the mixture was incubated in a water bath for 30 min at 37 °C. T he absorbance was read at 750 nm in a spectrophotometer (Thermo scientific, Genesys 10s UV-VIS, USA). C urve with gallic acid was performed with a range of concentration of 10–70 mg/L (Y = 0.0386x – 0.0081. The TPC was expressed as the milligram equivalent of gallic acid per gram of dried sample (mg GAE/g dried sample). The measures were done in triplicate.

### 2.6 Ferric reducing antioxidant power (FRAP)

The assay was done using the method described by Benzie and Strain (1996) with some modifications. 0.28 mL of diluted extract and 2.1 mL FRAP were added to eppendorf tubes and then incubated for 4 min at 37 °C before reading the absorbance at 593 nm using a quartz cell (Thermo scientific, Genesys 10s UV-VIS, USA). A calibration curve was performed with different concentrations of Trolox (0.1–0.8 mmol; Y = 0.0186x + 0.0546). *D ata* were expressed as mmolTE of Trolox equivalent per gram of dried sample material (mmol ET/g dried sample).

### 2.7 Instrumentation and chromatographic condition

Among the analytical techniques employed to separate and quantify the methylxanthines and the polyphenols in cocoa beans and cocoa by products, high-performance liquid chromatography (HPLC) is the most common technique, either in normal or in reverse phase (Ramli et al., 2001). The detection system most frequently employed are ultraviolet absorbance (Belšč ak et al., 2009; Ortega et al., 2010; Todorovic et al., 2015; Hernandez-Hernandez et al., 2018).

The HPLC analysis was carried out in a Thermo scientific ultimate 3000 equipped with a diode array detector, auto sampler and quaternary pump (Thermo Fisher Scientific Inc., Waltham, MA, USA). The separation of theobromine and caffeine were carried out using a Dionex (3 μm, 300 Å, 2.1 × 50 mm) at flow rate of 0.3 mL/min and a temperature of 25 °C. The mobile phase used was an aqueous solution of tetrahydrofuran (0.1% v/v) as eluent A and acetonitrile as solvent B in an isocratic run for 10 min. The separated analytes were monitored at 280 nm and were identified by comparing the retention times and spectral data to those of standards.

### 2.8 Statistical analysis

Data are presented as means ± standard deviations. Significant statistical differences between data sets were evaluated by one-way ANOVA with Tukey test using STATGRAPHICS® Centurion XVI. The level of p < 0.05 was considered statistically significant.

## 3 Results

The characterization of the plant material was carried out using 9 quantitative descriptors (Table 2), in which significant differences (p < 0.05) were found in the physical characteristics between all the evaluated genotypes. UTVE01 and CCN51 had the highest values of WEI_FRU, WEI_SEM, NUM_SEM, LON_FRU, DIA_FRU followed by UTLM02, UTGC01 and UTLP02. Highlighting the clone UTVE01 as the one with best results based on the number of seeds and grain index, thus allowing it to generate higher yields.

**Table 2:** Qualitative descriptors of genotypes from Chaparral municipality (Tolima).

Material	UTLP02	UTGC01	UTLM02	UTVE01	CCN51
WEI_FRU	448.92 ± 21.60 ^ab^	401.77 ± 40.94 ^ab^	369.36 ± 41.72^a^	533.50 ± 32.37^b^	778.66 ± 31.49^c^
LON_FRU	153.92 ± 4.93 ^ab^	168.71 ± 9.38^b^	145.55 ± 4.18^a^	163.31 ± 2.33 ^ab^	155.60 ± 1.69 ^ab^
DIA_FRU	81.15 ± 1.67 ^ab^	75.57 ± 2.72^a^	74.36 ± 2.68^a^	80.08 ± 1.15 ^ab^	84.90 ± 0.57 ^ab^
WEI_SE	49.60 ± 11.23^a^	45.25 ± 4.82^a^	78.19 ± 15.39 ^ab^	100.88 ± 5.82^b^	98.38 ± 3.0^b^
NUM_SE	13.69 ± 0.46^a^	32.14 ± 4.88 ^bc^	23.91 ± 2.54^b^	26.08 ± 1.71^b^	37.3 ± 1.53^c^
IND_SE	1 .4	1. 7	1. 5	1. 2	1. 6
WEI_HUS	324.06 ± 20.55 ^ab^	331.44 ± 43.86 ^ab^	235.72 ± 26.06^a^	400.78 ± 23.16^b^	696.08 ± 33.11^c^
SHE_THI	13.54 ± 0.48 ^abc^	11.86 ± 0.26^a^	12.36 ± 1.15 ^ab^	15.69 ± 0.60^c^	14.90 ± 0.31 ^bc^
L/D_SEM	1.966	2.0	1.818	1.833	1.699

N = 50 ± Standard deviation; results within a row followed by the same letters are not significantly different at the 0.05 level according to ANOVA.

Regarding the results obtained in the content of total polyphenols and antioxidant activity, a TPC ranging from 44.51 ± 0.90 to 106.77 ± 5.21 mg GAE/g was found with significant difference (p < 0.05) among the clones evaluated. The antioxidant activity of the cocoa bean extracts based on the FRAP test (Table 3), indicates that clone UTGC01 presented the highest antioxidant activity with 56.17 ± 1.764 mmolTE/g.

**Table 3:** Total phenolic content (TPC), antioxidant activity (FRAP), methylxanthine content and T/C ratio.

Variety	UTLP02	UTGC01	UTLM02	UTVE01	CCN51
TPC (mg GAE/g)	44.51 ± 0.90^a^	106.77 ± 5.21^d^	77.96 ± 3.94^e^	62.96 ± 1.29^b^	95.41 ± 2.50^c^
FRAP (mmol TE/g)	27.13 ± 0.14^a^	56.17 ± 1.74^c^	49.24 ± 2.86^b^	31.18 ± 0.54^a^	52.12 ± 4.71^bc^
TB (mg/g)	6.02 ± 0.04^c^	6.41 ± 0.06^d^	7.12 ± 0.15^e^	5.63 ± 0.04^b^	5.35 ± 0.09^a^
CF (mg/g)	0.51 ± 0.01^a^	1.08 ± 0.02^c^	0.91 ± 0.03^b^	2.31 ± 0.03^d^	0.52 ± 0.06^a^
T/C RATIO	12.14 ± 0.42^e^	5.94 ± 0.18^b^	7.72 ± 0.40^c^	2.44 ± 0.03^a^	10.11 ± 0.99^d^

Results are expressed as mean ± SD (n = 3). Results within a row followed by the same letters are not significantly different at the 0.05 level according to ANOVA.

**Figure 1:**
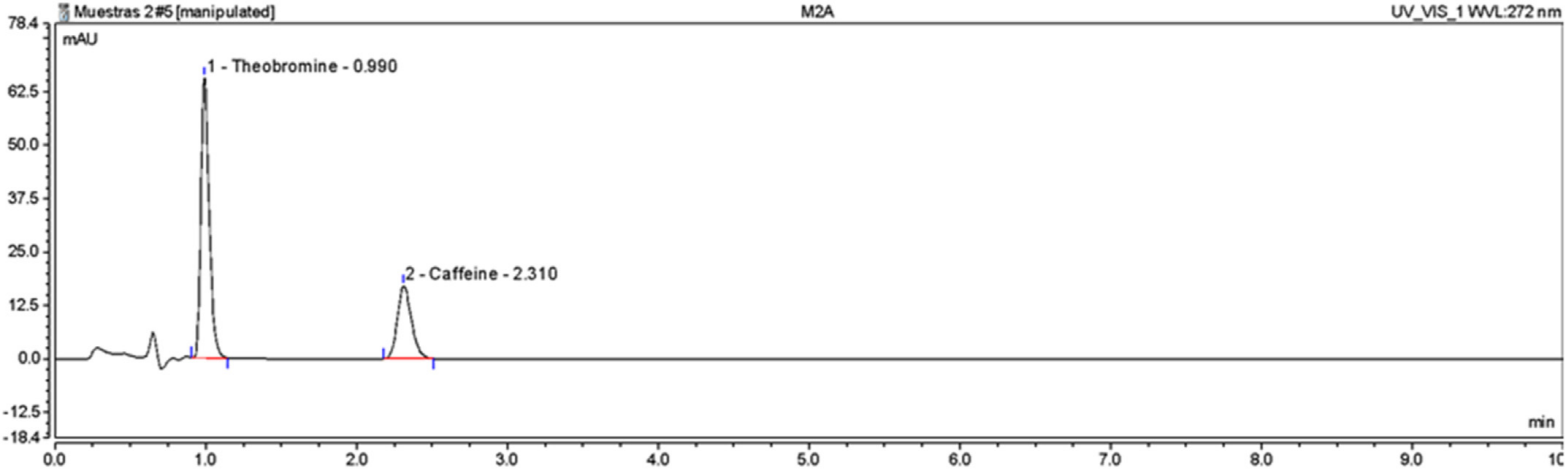
Theobromine and caffeine chromatogram of UTVE01 genotype.

The methylxanthines content was evaluated using the HPLC method (Figure 1). As seen in Figure 3, the UTVE 01 genotype presented the lower methylxanthine ratio 2.44, whereby it could be classified as C riollo genotype, UTGC01 and UTLM02 were found to be T rinitario, while UTLP02 and CCN51 are F orasteros. This classification is based on previous studies that have reported that TB was found in higher concentration than CF in cocoa beans (Brunetto et al., 2007). In the present work it was found that the TB content ranged between 7.12 ± 0.15 and 5.35 ± 0.09 mg/g while the CF ranged from 2.31 ± 0.03 to 0.51 ± 0.01 mg/g of defatted cocoa. Significant differences (p < 0.05) were found in the content of both methylxanthines (Table 3).

## 4 Discussion

The genotypes analyzed were compared against CCN51 clone values which have the highest expansion in production for Colombia and other Latin American countries. Currently, there is controversy around this clone, because genetic diversity and the production of fine or flavour cocoa could be affected if the new plantations are based solely on the high yield and resistances (Pigache and Bainville, 2007). At the morphological level, different variables were observed, not only quantitative during the process, but also qualitative (data not shown), such as the purple color presented by different genotypes at seed level. Enrí quez, 1993, refers to the color as a genetic differentiator between individuals. This difference was observed between the UTVE01 material with the rest of the genotypes, which was characterized by seeds of light cream color, unlike the other clones which presented purple seeds in different intensities (Table 4), which can be characterized as F orastero or T rinitario types (Barros, 1970). This genetic difference was confirmed at the level of the TB/CF ratio of each material.

**Table 4:** Descriptors from the beans of the genotypes evaluated.

Genotype	Bean color	Bean Shape
UTLP02	dark violet	oblong
UTGC01	dark violet	Irregular
UTLM02	dark violet	oval
UTVE01	cream	oblong
CCN51	dark violet	oval

One of the most important characteristics when evaluating a genetic material is the seed index, which can be one of the most important variables for the market, in this parameter UTLP02, UTGC01 and UTLM02 had a value close to CCN51, while UTVE01 is below this value. It's well known that compared to other clones, CNN51 has higher yields, stronger resistances to pathogens and changes in climate conditions which is the reason this clone overcomes the evaluated varieties in the descriptors. However, despite these advantages, in the fine or flavour cocoa market, it is considered of less organoleptic quality (Boza et al*.*, 2014; Herrmann et al*.,*2014). Regarding the other descriptors like WEI_FRU, LON_FRU, DIA_FRU, WEI_SEM and NUM_SEM, the evaluated genotypes in some descriptors are below the values from CCN51. Our results from this genotype are similar to those reported in other studies for this clone in different regions of Colombia (Ló pez Hern á ndez et al., 2021). The UTVE01 and UTGC01 have similar results in the qualitative descriptors compared with the genotypes developed by the CATIE (Philips et al*.,* 2012). Whereby these two genotypes could be promising trees in regional breeding programs for their use commercially and become accepted in the national and international market, thus guaranteeing results that benefit the local producers (Rodriguez-Medina et al., 2019).

Nevertheless, it is important to consider that the yield of cocoa genotypes is a multifactorial process, where the genetics of the tree and the edaphoclimatic conditions interact. Therefore, a genetic variation with good yields in a specific area might not be the same in another (Akoa et al*.,* 2021; Ló pez Hern á ndez et al., 2021). Hence the importance of the search for new genotypes of cocoa trees with Criollo and Trinitario genetic characteristics have good adaptation and yields in the local producer areas.

The assessment of TPC found a range between 44.51 ± 0.90 to 106.77 ± 5.21 mg GAE/g within the ranges found by Pedan et al.(2018). Several studies have reported a high variation in the TPC of cocoa beans, these investigations attribute differences to variety, cultivar of cocoa, growing region, beans maturity, storage time after harvest and altitude (Elwers et al., 2009; Albertini et al., 2015; Oracz et al., 2015).

During the post-harvest steps, the cocoa beans undergo internal changes such that polyphenol content decreases during the process, especially in the drying and roasting steps (Alean et al., 2016; Ioannone et al*.*, 2015). Therefore, it is important to evaluate the TPC in raw cocoa beans for the identification of clones with a high content of polyphenols and antioxidants, considering the high interest in the pharmaceutical industry in these bioactive components, to which functional benefits are attributed. In this sense, the clones UTGC01 and CCN51 could be promising cultivars for this purpose.

**Figure 2:**
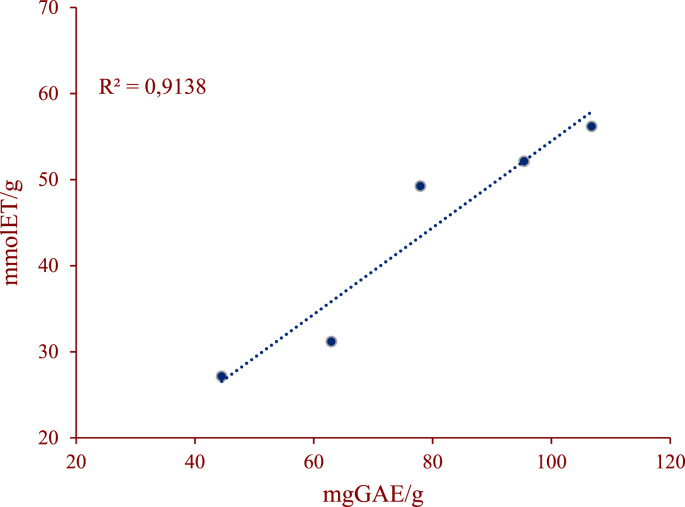
Relationship between the FRAP and TPC

The content of total polyphenols is directly related to the antioxidant activity of foods, which was measured by the FRAP method. Our results revealed that the antioxidant and TPC properties in beans from Chaparral-Tolima are comparable to those found in Ecuador and Ghana (Jonfia-Essien et al*.,* 2008; Albertini et al*.*, 2015). Furthermore, it is important to highlight the positive correlation between TPC and FRAP (r = 0.91) (Figure 2). Othman et al*.* (2007), also found a positive correlation (r = 0.764) between TPC and FRAP despite having worked with an ethanolic extract. These results are positive compared to the benefits attributed to the antioxidant capacity in human health, where the data found in the different regional genotypes, are comparative with antioxidants found in outstanding foods for their content, such as green tea (46.46 mg AG/g) (Rodrigo, 2019). Finally, these results suggest a genotypic difference between the cocoa clones evaluated, similar findings were reported by various authors (Ortega et al., 2010; Hernandez-Hernandez et al., 2018).

Currently more than 35 polyphenols have been reported in cocoa, these compounds are directly related with the high antioxidant activity (Rodriguez-Carrasco et al., 2018). The principal groups of polyphenols reported in cocoa are: catechins, procyanidins, anthocyanins and flavanols glycosides (Bordiga et al., 2015). It is well known that the polyphenolic profile changes according to the variety. In the case of Criollo genetics the amount of these compounds is 30%–60% less compared with T rinitario and F orastero (Martini et al., 2008), which explains the variations found between the genotypes evaluated in this study. However, it is difficult to compare the TPC and AA with those obtained in other investigations, mainly due to the differences in the solvent and extraction technique (Jonfia-Essien et al., 2008).

The ranges of TB and CF agreed with those found by Todorovic et al*.* (2015) and were close to Carrillo, Londoño & Gil (2013); Samaniego et al*.* (2020) As was expected the TB was the predominant alkaloid in the samples followed by CF. The content of these two methylxanthines have been reported varying depending on the edaphoclimatic conditions and the interaction with the genotype (Febrianto and Zhu, 2022). We corroborate that different genotypes and climatic zones have different TB and CF content.

**Figure 3:**
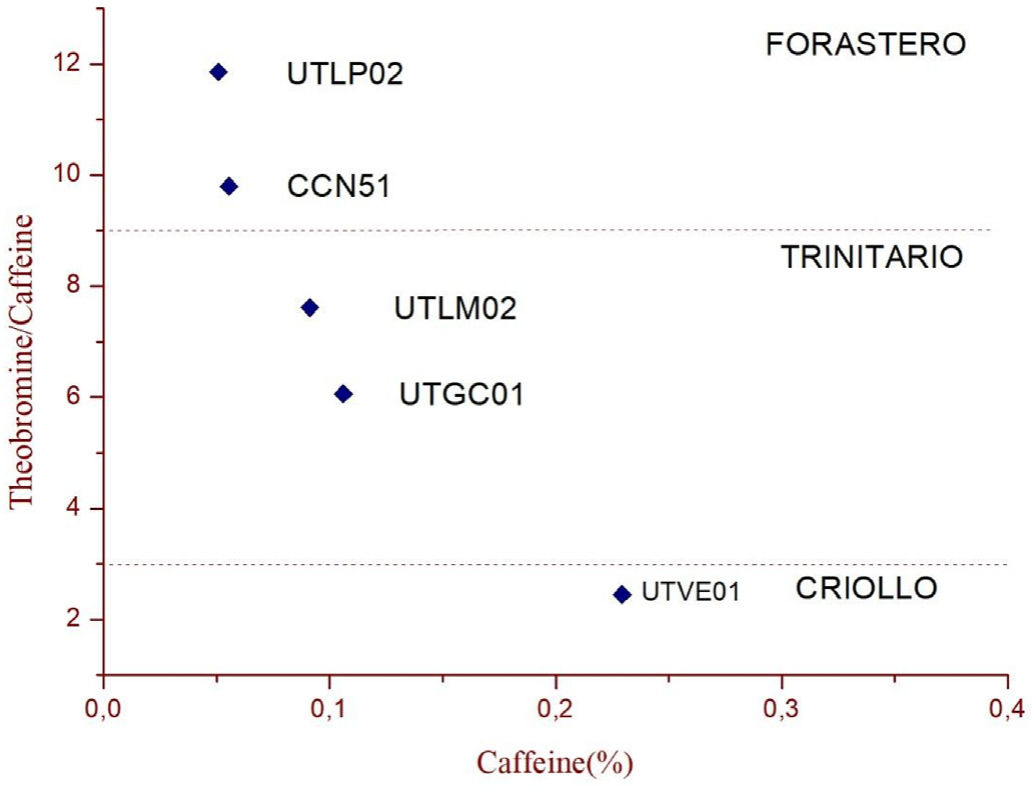
Relationship between the methylxanthine content and caffeine percentage.

The TB/CF relationship presented in Figure 3 shows that the ratio of these two methylxanthines is a good parameter to classify cocoa beans objectively and know the classification of the genotype. The C riollos genotypes are characterized by having less content of TB and more of CF, while the Forasteros have more TB and less CF and the T rinitario genotypes are in a midpoint. .This parameter has been used by authors to classify Forastero, Trinitario and Criollo beans from Venezuela, Colombia, and Ecuador. The TB/CF ratios of the evaluated genotypes found that only the clone UTVE01 could be classified as Criollo, UTGC1 and UTLM02 as T rinitario and UTPL02 like F orastero, this result is important since nowadays there is great interest in the Criollo and Trinitario varieties due to their aromatic and flavor profile resulting in better prices in the international market.

## 5 Conclusion

The present study classifies the UTVE01 genotype as Criollo type, which has superior characteristics at the morphoagronomic level as observed in the results, especially in the WEI_FRU and WEI_SEM, which is important for its yields. The T rinitario and C riollo genotypes found have a special interest in breeding programs of native cocoa varieties, to strengthen the production of fine or flavour cocoa. From the functional point of view genotypes like UTGC01, UTLM02 and CCN51 are attractive for the exploitation of their high antioxidants content, making these genotypes of great commercial interest, as part of nutritional products.

It is recommended to carry out further studies to characterize native genotypes since this project only covered specific areas of the Tolima department, it is possible to find more native materials already adapted to agro-climatic conditions with high possibilities in terms of production and flavour, to fulfill the market requirements.

## Declarations

### Author contribution statement

Juan. Borja Fajardo, Heidi B. Horta Tellez: Performed the experiments; Analyzed and interpreted the data; Wrote the paper.

Giann C. Penaloza Atuesta, Jonh J. Mendez Arteaga: Conceived and designed the experiments.

Angélica P. Sandoval Aldana: Analyzed and interpreted the data; Contributed reagents, materials, analysis tools or data; Wrote the paper.

### Funding statement

This work was supported by the government of Tolima and Universidad del Tolima [agreement No. 2078] entitled “Development of technological solutions for cacao cocoa production and processing” with internal code 130617.

### Data availability statement

Data will be made available on request.

### Declaration of interests statement

The authors declare no conflict of interest.

### Additional information

No additional information is available for this paper.
